# Cancer development in patients with COPD: a retrospective analysis of the National Health Insurance Service-National Sample Cohort in Korea

**DOI:** 10.1186/s12890-020-01194-8

**Published:** 2020-06-15

**Authors:** Song Vogue Ahn, Eunyoung Lee, Bumhee Park, Jin Hee Jung, Ji Eun Park, Seung Soo Sheen, Kwang Joo Park, Sung Chul Hwang, Jae Bum Park, Hae-Sim Park, Joo Hun Park

**Affiliations:** 1 Department of Health Convergence, Ewha Womans University, Seoul, South Korea; 2 Department of Biomedical Informatics, Ajou University School of Medicine, Suwon, South Korea; 3 Office of Biostatistics, Medical Research Collaborating Center, Ajou Research Institute for Innovative Medicine, Ajou University Medical Center, Suwon, South Korea; 4 Department of Pulmonary and Critical Care Medicine, Ajou University School of Medicine, Worldcup road 164, Suwon, Gyeonggi-do 16499 South Korea; 5 Department of Occupational and Environmental Medicine, Ajou University School of Medicine, Suwon, South Korea; 6 Department of Allergy and Clinical Immunolgy, Ajou University School of Medicine, Suwon, South Korea

**Keywords:** COPD, Cancer, Smoking

## Abstract

**Background:**

COPD is a well-known risk factor for lung cancer, independent of smoking behavior. By investigating the retrospective National Health Insurance Service-National Sample Cohort (NHIS-NSC) in Korea, this study attempted to prove the hypothesis that COPD is a risk factor for major cancers developing outside of the lungs. We also aimed to investigate the environmental factors associated with the development of lung cancer in COPD patients.

**Methods:**

This study analyzed data from the NHIS-NSC over a 12-year period. Among the 514,795 subjects in the NHIS-NSC, 16,757 patients who were diagnosed with any cancer from 2002 to 2003 were excluded. This cohort enrolled six arms consisting of never-smokers without COPD (*N* = 313,553), former smokers without COPD (*N* = 41,359), smokers without COPD (*N* = 112,627), never-smokers with COPD (*N* = 7789), former smokers with COPD (*N* = 1085), and smokers with COPD (*N* = 2677).

**Results:**

Incident rate of lung cancer per 100,000 person-year was higher according to smoking and COPD (216 in non-COPD and 757 in COPD among never-smokers, 271 in non-COPD and 1266 in COPD among former smokers, 394 in non-COPD and 1560 in COPD among smokers, *p* <  0.01). Old age, male sex, lower BMI, low exercise level, history of diabetes mellitus, smoking, and COPD were independent factors associated with the development of lung cancer (*p* <  0.01). Multi-variable analyses showed that COPD, regardless of smoking status, contributed to the development of lung cancer, and colorectal cancer and liver cancer among other major cancers (*p* <  0.01).

**Conclusion:**

Our data suggested that COPD was an independent risk factor for the development of lung cancer, and colorectal cancer and liver cancer among other major cancers in the Korean population, regardless of smoking status.

## Background

Chronic obstructive pulmonary disease (COPD) outpaces other major diseases as a cause of mortality throughout the entire world and is expected to rank third among all causes of death by 2020 [1–3].

Many co-morbidities accompanying COPD influence the major outcomes of COPD [2, 4, 5]. Interestingly, COPD is a well-known risk factor for the development of lung cancer, independent of smoking behavior [6–8]. Therefore, many studies have evaluated the relationship between COPD and lung cancer [6–9]. The pathologic mechanism contributing to the development of lung cancer in COPD has been explained by telomere shortening, chronic inflammation, the increased expression of growth factors in COPD lungs, and oxidant-induced DNA damage resulting in mutations and carcinogenesis [8, 10, 11]. However, it has not been reported yet whether COPD can be a risk factor for cancers developing outside of the lungs despite the evidence that systemic inflammation is the characteristic feature of COPD [2, 5]. In addition to smoking, radiation, exposure to carcinogens, such as asbestos and radon, air pollution, etc., are risk factors for the development of lung cancer [12, 13]. Nevertheless, most environmental risk factors for lung cancer, except for smoking exposure, require further validation.

Therefore, the authors hypothesized that the process of tumorigenesis in COPD may not be limited to the lungs but can also affect the body systemically, leading to the development of major cancers outside the lungs. Hence, this study attempted to prove the hypothesis that COPD is a risk factor for, not only lung cancer but also other major cancers by investigating a cohort in the National Health Insurance Service–National Sample Cohort (NHIS-NSC). The current study also further sought to examine the environmental factors associated with the development of lung cancer in COPD patients.

## Methods

### Study population

**Fig. 1:**
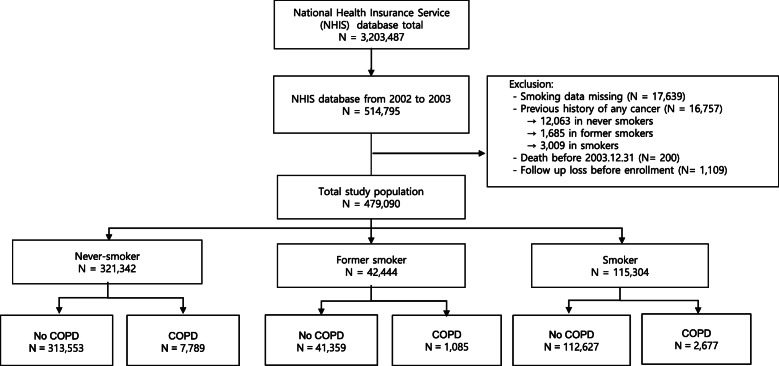
Flow diagram of this study

This study analyzed data from the National Health Insurance Service-National Sample Cohort (NHIS-NSC). The NHIS-NSC is a population-based cohort established by the National Health Insurance Service (NHIS) in South Korea [14]. The NHIS is a single-payer health insurance system in South Korea that covers the entire South Korean population (approximately 48 million in 2003). The NHIS provides biennial health screening to all insured adults aged 40 years or older. The NHIS-NCS database consists of 514,795 participants who were aged between 40 and 79 in 2002 and underwent health screening programs in 2002 or 2003; 2002 for participants born in an even year and 2003 for participants born in an odd year (Fig. 1).

**Fig. 2:**
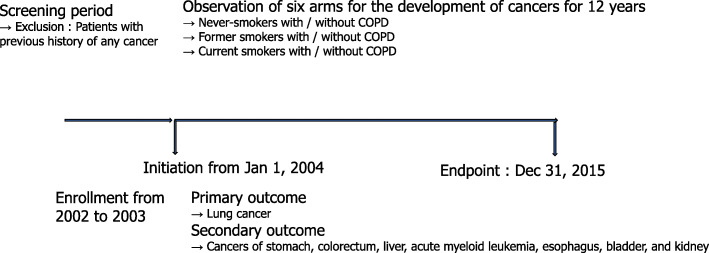
Study design

After excluding 16,757 patients (12,063 never smokers, 1685 former smokers, and 3009 smokers) with previous histories of cancer diagnoses before January 1, 2004, classified by the International Classification of Diseases 10th revision (ICD-10) codes for cancer diagnoses or questionnaires on previous medical history, 321,342 never-smokers, 42,444 former smokers, and 115,304 smokers were included in the final study cohort (Fig. 1). Finally, the cohort consisted of six arms, never-smokers without COPD (*N* = 313,553), former smokers without COPD (*N* = 41,359), smokers without COPD (*N* = 112,627), never-smokers with COPD (*N* = 7789), former smokers with COPD (*N* = 1085), and smokers with COPD (*N* = 2677) (Fig. 1). We investigated the development of new cancers, including lung cancer, stomach cancer, colorectal cancer, liver cancer, acute myeloid leukemia (AML), esophageal cancer, bladder cancer, and kidney cancer for a 12-year period from January 1, 2004, to December 31, 2015 (Figs. 1 and 2).

### Definition and covariates

COPD was identified by the combination of J43-J44 ICD-10 codes for COPD and use of the following medications for COPD (≥ 4 times in 2 years): long-acting muscarinic antagonists (LAMA), long-acting beta-2 agonists (LABA), LAMA + LABA, inhaled corticosteroids (ICS) + LABA, triple therapy (LAMA + LABA + ICS), short-acting muscarinic antagonists (SAMA), short-acting beta-2 agonists (SABA), phosphodiesterase-4 (PDE-4) inhibitors, mucolytics, or theophylline [15, 16].

Detailed history of smoking (smoking amount, duration, and non-smoking period) and exercise status (intensity and frequency per week) were evaluated by self-administered questionnaires at baseline in 2002 or 2003. Former smokers were defined as smokers whose smoking cessation period was 1 year or more at enrollment [17]. Data on body mass index (body weight in kilograms divided by height in meters squared; kg/m^2^), systolic and diastolic blood pressure, fasting serum glucose, and fasting total cholesterol level measured at baseline were obtained.

### Main outcome measures

The primary outcome was the incidence of lung cancer. The secondary outcomes were the incidence of stomach cancer, colorectal cancer, liver cancer, AML, esophageal cancer, bladder cancer, and kidney cancer.

The outcome measures were ascertained by health insurance claims data in the NHIS from January 1, 2004, to December 31, 2015. The first incident event was only used in the analyses for participants with more than one event. ICD-10 codes were used to identify outcomes as follows: lung cancer (C33, C34), stomach cancer (C16), colorectal cancer (C18 - C20), liver cancer (C22), AML (C92.0), esophageal cancer (C15), bladder cancer (C67), and kidney cancer (C64 - C66).

### Statistical analyses

All values were expressed as mean ± standard deviation. Continuous and categorical variables were analyzed with one-way analyses of variance (ANOVA) and chi-square tests, respectively. Univariate Cox proportional hazards regression analyses were used to identify significant variables predicting the occurrence of an event individually (*p* <  0.01). Then, multivariable Cox proportional hazard models were performed to evaluate the independent effects of COPD on the development of cancer, adjusting for age, gender, hypertension, diabetes, body mass index, and exercise variables. Hazard ratios (HRs) with 95% confidence intervals (CIs) were calculated for the risk of lung cancer, stomach cancer, colorectal cancer, liver cancer, AML, esophageal cancer, bladder cancer, and kidney cancer.

A conservative threshold of *p* <  0.01 was determined to be significant, considering a large sample size of this study [18]. All analyses were performed using SAS version 9.4 (SAS Institute, Cary, NC, USA).

### Ethics statement

Informed written consent was obtained from all participants and the present study was approved by the Institutional Review Board of Ajou University Hospital (No. AJIRB-MED-EXP-17-167).

## Results

### Baseline characteristics of this cohort

The baseline characteristics of the NHIS-NSC participants are shown in Table 1.

**Table 1:** Baseline characteristics of the cohort

	Never smoker		Former smoker		Smoker		
	No COPD	COPD	No COPD	COPD	No COPD	COPD	*P*
Number	313,553 (65.45)	7789 (1.63)	41,359 (8.63)	1085 (0.23)	112,627 (23.51)	2677 (0.56)	
Age (years)	53.64 ± 9.62	62.23 ± 9.87	51.65 ± 9.12	62.43 ± 10.16	50.92 ± 8.74	61.29 ± 9.83	< 0.001
Gender (male), N (%)	107,393 (34.25)	2799 (35.94)	39,292 (95.00)	1005 (92.63)	106,695 (94.73)	2368 (88.46)	< 0.001
BMI (kg/m^2^)	24.08 ± 3.01	24.02 ± 3.39	24.31 ± 2.73	23.18 ± 3.05	23.73 ± 2.94	22.97 ± 3.28	< 0.001
< 20	23,181 (7.39)	847 (10.87)	2205 (5.33)	157 (14.47)	11,102 (9.86)	514 (19.20)	< 0.001
20 ≤ < 25	178,681 (56.99)	4071 (52.27)	22,958 (55.51)	642 (59.17)	65,174 (57.87)	1466 (54.76)	< 0.001
25 ≤ < 30	101,536 (32.38)	2556 (32.82)	15,247 (36.87)	265 (24.42)	34,045 (30.23)	633 (23.65)	< 0.001
30 ≤	10,155 (3.24)	315 (4.04)	949 (2.29)	21 (1.94)	2306 (2.05)	64 (2.39)	< 0.001
Systolic blood pressure (mmHg)	126.71 ± 18.55	130.61 ± 19.21	128.56 ± 17.16	130.07 ± 18.70	127.71 ± 17.55	128.88 ± 18.59	< 0.001
Diastolic blood pressure (mmHg)	97.28 ± 33.29	100.50 ± 38.18	99.56 ± 32.21	101.26 ± 44.08	100.40 ± 37.72	101.86 ± 34.78	< 0.001
Fasting serum glucose (mg/dL)	200.88 ± 38.50	201.56 ± 39.47	201.17 ± 37.52	196.79 ± 42.59	199.10 ± 38.39	195.80 ± 42.63	< 0.001
Total cholesterol (mg/dL)	313,553 (65.45)	7789 (1.63)	41,359 (8.63)	1085 (0.23)	112,627 (23.51)	2677 (0.56)	< 0.001
Exercise level							
Non exercise	187,422(60.81)	5387 (70.08)	16,159 (39.74)	619 (58.18)	59,485 (52.97)	1848 (69.42)	< 0.001
Once or twice a week	63,023 (20.45)	1078 (14.02)	13,986 (34.40)	239 (22.46)	33,756 (30.06)	450 (16.90)	
At least three times a week	57,778 (18.75)	1225 (15.90)	10,517 (25.86)	206 (19.36)	19,063 (16.97)	364 (13.67)	
Incident rate of lung cancer per 100,000 person year: average of six arms = 278	216	757	271	1266	394	1560	< 0.001
**Prevalence of cancer for 12 years, N (%)**							
Lung cancer (C33-C34)	7669 (2.44)	609 (7.82)	1263 (3.05)	129 (11.89)	4899 (4.35)	398 (14.87)	< 0.001
Stomach cancer (C16)	7979 (2.54)	310 (3.98)	1464 (3.54)	64 (5.90)	4170 (3.70)	143 (5.34)	< 0.001
Colorectal cancer (C18-C20)	12,126 (3.87)	505 (6.48)	1847 (4.47)	65 (5.99)	4783 (4.25)	203 (7.58)	< 0.001
Liver cancer (C22)	12,681 (4.04)	446 (5.73)	2091 (5.06)	81 (7.47)	6131 (5.44)	188 (7.02)	< 0.001
AML (C92.0)	181 (0.06)	5 (0.06)	19 (0.05)	1 (0.09)	74 (0.07)	3 (0.11)	0.600
Esophageal cancer (C15)	463 (0.15)	29 (0.37)	124 (0.30)	3 (0.28)	474 (0.42)	15 (0.56)	< 0.001
Bladder cancer (C67)	2367 (0.75)	100 (1.28)	426 (1.03)	18 (1.66)	1083 (0.96)	39 (1.46)	< 0.001
Kidney cancer (C64-C66)	1740 (0.55)	58 (0.74)	276 (0.67)	12 (1.11)	729 (0.65)	24 (0.90)	< 0.001
**Past medical history, N (%)**							
Hypertension	29,436 (9.39)	1257 (16.14)	3334 (8.06)	143 (13.18)	5974 (5.30)	282 (10.53)	< 0.001
Diabetes mellitus	12,904 (4.12)	539 (6.92)	1949 (4.71)	65 (5.99)	4559 (4.05)	173 (6.46)	< 0.001

*Definition of abbreviations*: *AML* Acute myeloid leukemia, *BMI* Body mass index, *COPD* Chronic obstructive pulmonary disease

Among 514,795 subjects in the NHIS-NSC database, this study enrolled six arms consisting of never-smokers without COPD (*N* = 313,553, males = 34.25%), former smokers without COPD (*N* = 41,359, males = 95.00%), smokers without COPD (*N* = 112,627, males = 94.73%), never-smokers with COPD (*N* = 7789, males = 35.94%), former smokers with COPD (*N* = 1085, males = 92.63%), and smokers with COPD (*N* = 2677, males = 88.46%) selected by COPD ICD 10 codes, history of COPD medications, and excluding patients with previous histories of any cancer (Fig. 1).

During the 12-year period, lung cancer developed much higher in never-smokers with COPD (7.82%), former smokers with COPD (11.89%), and smokers with COPD (14.87%) than in never-smokers without COPD (2.44%) (*p* <  0.001). Incident rate of lung cancer per 100,000 person-year was higher according to smoking and COPD (216 in non-COPD and 757 in COPD among never-smokers, 271 in non-COPD and 1266 in COPD among former smokers, 394 in non-COPD and 1560 in COPD among smokers, *p* <  0.001).

The prevalence of lung cancer, stomach cancer, colorectal cancer, liver cancer, esophageal cancer, bladder cancer, and kidney cancer was higher according to smoking history and COPD diagnosis (*p* <  0.001).

### Risk factors for the development of lung cancer

Univariate cox regression analysis found that older age, male sex, lower BMI, history of hypertension, history of diabetes mellitus, exercise level, COPD diagnosis, and smoking history were associated with the development of lung cancer (*p* <  0.001) (Table 2). Multi-variable Cox regression analyses showed that older age, male sex, lower BMI, exercise level, COPD diagnosis, and smoking history were independently associated with the development of lung cancer (*p* <  0.01) (Table 2).

**Table 2:** Risk factors for the development of lung cancer

	Cox regression analysis			
	Univariate Hazard ratio (95% CI)	*p*-value	Multivariate Hazard ratio (95% CI)	*p*-value
Age (years)	1.072 (1.070–1.074)	< 0.001	1.074 (1.072–1.076)	< 0.001
Male (vs female)	1.866 (1.803–1.931)	< 0.001	1.789 (1.717–1.864)	< 0.001
BMI (kg/m^2^)		< 0.001		< 0.001
< 20	1.661(1.582–1.744)		1.323 (1.256–1.393)	
20 ≤ < 25	Reference		Reference	
25 ≤ < 30	0.852 (0.769–0.945)		0.885 (0.795–0.985)	
30 ≤	0.446 (0.396–0.501)		0.837 (0.705–0.994)	
History of hypertension	1.414(1.344–1.488)	< 0.001	0.990(0.939–1.044)	0.718
History of diabetes mellitus	1.579(1.477–1.688)	< 0.001	1.186(1.108–1.269)	< 0.001
COPD diagnosis	3.752(3.530–3.988)	< 0.001	2.046(1.922–2.177)	< 0.001
Exercise level		< 0.001		< 0.001
Never Exercise	1.178 (1.129–1.229)		1.145 (1.095–1.198)	
1–2 times a week	0.888 (0.816–0.966)		1.010 (0.925–1.103)	
≥ 3 times a week	Reference		Reference	
Smoking status		< 0.001		< 0.001
Never smoker	Reference		Reference	
Former smoker	1.277(1.205–1.352)		1.108(1.042–1.178)	
Current smoker	1.860(1.797–1.926)		1.689(1.622–1.759)	

*Definition of abbreviations*: *BMI* Body mass index, *COPD* Chronic obstructive pulmonary disease, * = statistically significant hazard ratio (*p*-value < 0.01)

### Risk factors for the development of other major cancers including stomach cancer, colorectal cancer, liver cancer, esophageal cancer, and bladder cancer

Smoking was independently associated with the development of stomach cancer, colorectal cancer, liver cancer, esophageal cancer, and bladder cancer, and exercise level was independently associated with the development of stomach cancer and liver cancer (*p* <  0.01) (Supplement table 1, 2, 3, 4 and 5).

### COPD as a risk factor for the development of major cancers

Multi-variable cox regression analyses were performed in three models: Model 1 (adjusted for age and sex), Model 2 (Model 1 + an additional adjustment for history of hypertension and history of diabetes mellitus), Model 3 (Model 2 + an additional adjustment for BMI and exercise). The multi-variable Cox regression analyses demonstrated that COPD in never-smokers, former smokers, and smokers contributed to the development of lung cancer in all three models (Model 3: COPD in never-smokers, HR = 2.046; COPD in former smokers, HR = 2.267; COPD in smokers, HR = 3.456 (all *p*-values < 0.001) (Table 3). In addition, COPD in never-smokers, former smokers, and current smokers contributed to the development of colorectal cancer and liver cancer among other major cancers (all *p*-values < 0.001) (Tables 3 and 4).

**Table 3:** COPD as a risk factor for the development of major cancers by multi-variate cox regression analyses

	Hazard Ratio (95% CI)					
	Model 1 Hazard ratio (95% CI)	*p*-value	Model 2 Hazard ratio (95% CI)	*p*-value	Model 3 Hazard ratio (95% CI)	*p*-value
Lung cancer						
Never smoker without COPD	Reference		Reference		Reference	
Former smoker without COPD	1.100 (1.035–1.170)	0.002	1.100 (1.035–1.169)	0.002	1.108 (1.043–1.178)	< 0.001
Smoker without COPD	1.752 (1.683–1.823)	< 0.001	1.749 (1.681–1.821)	< 0.001	1.689 (1.622–1.759)	< 0.001
Never smoker with COPD	2.086 (1.960–2.220)	< 0.001	2.086 (1.960–2.220)	< 0.001	2.046 (1.922–2.177)	< 0.001
Former smoker with COPD	2.295 (2.105–2.503)	< 0.001	2.294 (2.104–2.502)	< 0.001	2.267 (2.079–2.472)	0.001
Current smoker with COPD	3.654 (3.396–3.932)	< 0.001	3.650 (3.392–3.927)	< 0.001	3.456 (3.211–3.720)	< 0.001
Stomach cancer						
Never smoker without COPD	Reference		Reference		Reference	
Former smoker without COPD	1.105 (1.042–1.172)	0.001	1.104 (1.041–1.171)	0.001	1.111 (1.048–1.178)	< 0.001
Smoker without COPD	1.249 (1.198–1.302)	< 0.001	1.249 (1.197–1.302)	< 0.001	1.289 (1.184–1.289)	< 0.001
Never smoker with COPD	1.067 (0.976–1.167)	0.154	1.067 (0.976–1.167)	0.155	1.056 (0.966–1.155)	0.231
Former smoker with COPD	1.179 (1.060–1.312)	0.002	1.178 (1.060–1.311)	0.003	1.174 (1.055–1.306)	0.003
Current smoker with COPD	1.333 (1.208–1.470)	< 0.001	1.332 (1.208–1.470)	< 0.001	1.305 (1.183–1.440)	< 0.001
Colorectal cancer						
Never smoker without COPD	Reference		Reference		Reference	
Former smoker without COPD	1.045 (0.992–1.102)	0.096	1.043 (0.990–1.099)	0.113	1.046 (0.992–1.102)	0.095
Smoker without COPD	1.086 (1.046–1.128)	< 0.001	1.089 (1.048–1.131)	< 0.001	1.088 (1.047–1.130)	< 0.001
Never smoker with COPD	1.277 (1.197–1.374)	< 0.001	1.277 (1.186–1.374)	< 0.001	1.273 (1.183–1.370)	< 0.001
Former smoker with COPD	1.335 (1.221–1.460)	< 0.001	1.332 (1.218–1.456)	< 0.001	1.331 (1.217–1.455)	< 0.001
Current smoker with COPD	1.387 (1.278–1.505)	< 0.001	1.390 (1.281–1.509)	< 0.001	1.385 (1.276–1.503)	< 0.001
Liver cancer						
Never smoker without COPD	Reference		Reference		Reference	
Former smoker without COPD	1.034 (0.985–1.086)	0.181	1.031 (0.982–1.083)	0.219	1.040 (0.990–1.093)	0.116
Smoker without COPD	1.168 (1.128–1.209)	< 0.001	1.168 (1.128–1.210)	< 0.001	1.162 (1.122–1.204)	< 0.001
Never smoker with COPD	1.229 (1.139–1.322)	< 0.001	1.228 (1.138–1.325)	< 0.001	1.217 (1.128–1.313)	< 0.001
Former smoker with COPD	1.271 (1.162–1.391)	< 0.001	1.266 (1.157–1.386)	< 0.001	1.266 (1.157–1.386)	< 0.001
Current smoker with COPD	1.435 (1.321–1.560)	< 0.001	1.434 (1.320–1.559)	< 0.001	1.415 (1.302–1.537)	< 0.001

Model 1: adjusted for age and sex

Model 2: Model 1 + additional adjustment for history of hypertension, and history of diabetes mellitus

Model 3: Model 2 + additional adjustment for BMI and Exercise

*Definition of abbreviations*: *BMI* Body mass index, *COPD* Chronic obstructive pulmonary disease, * = statistically significant hazard ratio (*p*-value < 0.01)

**Table 4:** COPD as a risk factor for the development of other cancers by multi-variate cox regression analyses

	Hazard Ratio (95% CI)					
	Model 1 Hazard ratio (95% CI)	*p*-value	Model 2 Hazard ratio (95% CI)	*p*-value	Model 3 Hazard ratio (95% CI)	*p*-value
AML						
Never smoker without COPD	Reference		Reference		Reference	
Former smoker without COPD	0.617 (0.382–0.998)	0.049	0.616 (0.381–0.996)	0.048	0.632 (0.391–1.022)	0.061
Smoker without COPD	0.922 (0.684–1.242)	0.593	0.925 (0.686–1.246)	0.608	0.914 (0.678–1.233)	0.556
Never smoker with COPD	1.064 (0.544–2.083)	0.856	1.064 (0.544–2.082)	0.857	1.047 (0.535–2.049)	0.895
Former smoker with COPD	0.657 (0.289–1.494)	0.316	0.655 (0.288–1.491)	0.314	0.661 (0.291–1.505)	0.324
Current smoker with COPD	0.981 (0.473–2.037)	0.959	0.984 (0.474–2.043)	0.965	0.957 (0.460–1.989)	0.905
Esphageal cancer						
Never smoker without COPD	Reference		Reference		Reference	
Former smoker without COPD	1.303 (1.061–1.600)	0.012	1.303 (1.061–1.599)	0.012	1.304 (1.062–1.601)	0.011
Smoker without COPD	2.082(1.810–2.395)	< 0.001	2.080 (1.808–2.394)	< 0.001	1.968 (1.709–2.267)	< 0.001
Never smoker with COPD	0.973 (0.722–1.312)	0.858	0.973 (0.722–1.312)	0.858	0.945 (0.700–1.274)	0.708
Former smoker with COPD	1.268 (0.885–1.817)	0.197	1.268 (0.884–1.817)	0.197	1.231 (0.859–1.765)	0.257
Current smoker with COPD	2.026 (1.460–2.810)	< 0.001	2.024 (1.459–2.808)	< 0.001	1.859 (1.338–2.583)	< 0.001
Bladder cancer						
Never smoker without COPD	Reference		Reference		Reference	
Former smoker without COPD	1.090 (0.977–1.216)	0.121	1.088 (0.975–1.214)	0.129	1.089 (0.976–1.215)	0.127
Smoker without COPD	1.131 (1.044–1.225)	0.003	1.136 (1.049–1.231)	0.002	1.142 (1.053–1.237)	0.001
Never smoker with COPD	1.145 (0.973–1.347)	0.104	1.145 (0.973–1.347)	0.103	1.146 (0.974–1.348)	0.101
Former smoker with COPD	1.248 (1.027–1.516)	0.026	1.246 (1.026–1.514)	0.027	1.247 (1.027–1.516)	0.026
Current smoker with COPD	1.295 (1.081–1.550)	0.005	1.301(1.087–1.558)	0.004	1.308 (1.092–1.566)	0.004
Kidney cancer						
Never smoker without COPD	Reference		Reference		Reference	
Former smoker without COPD	1.002 (0.875–1.148)	0.975	0.998 (0.871–1.143)	0.974	0.999 (0.873–1.144)	0.992
Smoker without COPD	1.054 (0.956–1.163)	0.288	1.066 (0.967–1.176)	0.198	1.075 (0.974–1.185)	0.152
Never smoker with COPD	1.076 (0.870–1.331)	0.499	1.076 (0.870–1.330)	0.501	1.077 (0.871–1.332)	0.493
Former smoker with COPD	1.079 (0.839–1.386)	0.555	1.073 (0.835–1.379)	0.581	1.076 (0.838–1.383)	0.565
Current smoker with COPD	1.135 (0.899–1.432)	0.287	1.147 (0.909–1.447)	0.247	1.157 (0.917–1.461)	0.219

Model 1: adjusted for age and sex

Model 2: Model 1 + additional adjustment for history of hypertension, and history of diabetes mellitus

Model 3: Model 2 + additional adjustment for BMI and Exercise

*Definition of abbreviations*: *AML* Acute myeloid leukemia, *BMI* Body mass index, *COPD* Chronic obstructive pulmonary disease, * = statistically significant hazard ratio (*p*-value < 0.01)

## Discussion

Our study provides a comprehensive analysis of COPD as a risk factor for major cancers in the Korean population. Our data revealed that COPD in the Korean population was an independent risk factor contributing to the development of lung cancer, and colorectal cancer and liver cancer among other major cancers, irrespective of smoking status. In this analysis of a national cohort representative of the Korean population with up to 12 years of follow-up, multi-variable analysis demonstrated that male gender, lower BMI, history of diabetes mellitus, and exercise level, along with smoking status and COPD diagnosis, were independent risk factors for the development of lung cancer.

Our study presents several interesting findings.

First, our data showed that COPD diagnosis was independently associated with the occurrence of lung cancer by all models analyzed by Cox regression analyses, suggesting that COPD per se contributes to the development of lung cancer, irrespective of smoking behavior. Studies based on Korean National Health and Nutrition Examination Survey also showed that advanced age, male gender, low income, pulmonary tuberculosis, and asthma were independent risk factors for COPD in non-smokers [19, 20]. The incident rate of lung cancer per 100,000 person-year was higher than the previous report on general Korean population [21]. This higher rate of lung cancer could be explained by the average older age of this cohort (higher than 50 years old in all six arms), because of which this cohort cannot represent general Korean population.

The association between COPD and lung cancer has been explained by several mechanisms [22–24]. Repeated injury and repair by chronic inflammation and frequent exacerbations in COPD may result in tissue injury and DNA damage, leading to malignant cell transformation and the development of lung cancer [22]. Multiple genetic factors may explain the link between the development of COPD and lung carcinogenesis [23, 24]. However, there is an opposing perspective that the pathologic processes of COPD and lung cancer appear to be different, since features of COPD include destruction and apoptosis, whereas lung cancer is characterized by unrestrained proliferation and lack of apoptosis [9, 25].

Recent bodies of clinical evidence have suggested that emphysema and severe airflow obstruction increased the risk of lung cancer beyond the effect of smoking [26, 27]. Several pathological mechanisms, including premature aging, genetic predisposition, and epigenetic changes, have been proposed to explain the carcinogenesis in emphysematous lungs [10, 28]. In a clinical trial in a large Veterans Affairs patient cohort, statins were shown to be protective against the development of lung cancer, reducing the incidence of lung cancer over 50% [29].

Second, our data found that the prevalence of stomach cancer, colorectal cancer, and liver cancer was higher according to smoking and COPD diagnoses. Multi-variable analyses demonstrated an independent association between COPD, and colorectal cancer and liver cancer among other major cancers. Theoretically, the spillover of aberrant inflammation in COPD can lead to systemic consequences, such as carcinogenesis in other organs. Our study supports our original hypothesis that COPD is an independent risk factor for the development of some major cancers occurring outside the lungs. A recent study demonstrated the intimate relationship between malignant cells and their inflammatory microenvironment [30]. Inflammation is increased in COPD and experiments with anti-inflammatory treatments, such as Nrf2 activators and statins, showed the potential to inhibit the proliferation of cancer cells by reducing inflammation [31]. To our knowledge, no study has ever reported any link between COPD and major cancers occurring outside the lung. Hence, these findings should be further validated with other big cohorts.

Third, this cohort study identified environmental factors including low exercise level and lower BMI, as independent risk factors contributing to the development of lung cancer, indicating that multidisciplinary approaches are required for the prevention of lung cancer in COPD.

Our finding that a reduced BMI was independently linked to the development of lung cancer contradicts the conventional notion that obesity is pathogenically linked to carcinogenesis [32, 33]. Several studies highlighted an obesity paradox suggesting that obesity may be protective and associated with reduced lung cancer mortality after surgery or chemotherapy [32, 34, 35]. Although the mechanisms behind this paradoxical relationship are not fully understood, anti-tumor adipokines, anti-tumor energy reserve, metabolic fitness, relative lack of sarcopenia, etc., have been suggested as potential biological mechanisms to explain this obesity paradox [34]. Most studies, however, have focused on the mortality of patients with lung cancer after surgery or chemotherapy [32, 34, 35]. Our study is different from previous studies on the prognosis after a lung cancer diagnosis because we investigated the development of cancers in a cancer-free cohort by a longitudinal design [32, 34, 35]. Our findings are supported by previous reports that emphysema characterized by lower BMI was a risk factor for lung cancer, although this study did not examine the phenotype of COPD in our cohort [36, 37].

However, a recent cohort study comparing 433 COPD patients with 279 healthy controls reported that obesity in COPD patients predicted higher lung cancer risk [36]. Therefore, further studies are required to investigate the controversial relationship between obesity and lung cancer.

Our finding that lower exercise levels were an independent risk factor for the development of lung cancer is supported by previous studies [38–40]. Some studies reported an approximate 25% reduction in lung cancer risk by higher levels of physical activities [38, 40]. Good adherence to established nutrition and physical activity through cancer prevention guidelines is recommended since significant reductions in overall cancer incidence and mortality were reported by these methods [39]. Epidemiologic studies have suggested that healthy lifestyle choices should be encouraged since controlling environmental factors, such as diet, BMI, and physical activity, can effectively lower the prevalence of cancers [39, 41]. Further prospectively designed research on environmental factors contributing to cancer development should provide valid scientific data from which to develop appropriate public health strategies.

We acknowledge several limitations of this study. First, defining COPD by pulmonary function test was not possible due to the lack of information. Second, the pathologic type of each cancer was not investigated because of limited information in the NHIS-NSC. Third, disease-specific risk factors were not controlled in some cancers, including infections by hepatitis B and C viruses in liver cancer and emphysema in lung cancer. Fourth, medication history was not taken into consideration in our analysis because the current study was not a randomized controlled trial. Fifth, air pollution and occupation were not investigated as causal factors for cancer development in our analysis. Sixth, some major cancers such as breast cancer and prostate cancer were not included in the analysis because data access was not available due to technological problems. Seventh, selection bias could be inherent in this study because the ratio of non-smoker COPD, women, and never-smokers was higher in this cohort than in general Korean population. The NHIS-NSC includes only those who participated in the health screening program provided by NHIS in South Korea. Hence, the unscreened population of Korea cannot be represented by this cohort.

## Conclusion

Our data demonstrated that a COPD diagnosis was an independent risk factor contributing to the development of lung cancer and, colorectal cancer and liver cancer among other major cancers, irrespective of smoking status in the Korean population. This study also suggested that old age, male gender, lower BMI, history of diabetes mellitus, and low exercise level, along with smoking status and COPD diagnosis were independent factors contributing to the development of lung cancer, suggesting that multidisciplinary approaches are required for the prevention of lung cancer in COPD patients.

## Supplementary information

**Additional file 1.** Table 1. Risk factors for the development of stomach cancer. Table 2. Risk factors for the development of colorectal cancer. Table 3. Risk factors for the development of liver cancer. Table 4. Risk factors for the development of esophageal cancer. Table 5. Risk factors for the development of bladder cancer.

## Data Availability

NHIS-NSC is an open and public data to which any researcher can get access through the website (https://nhiss.nhis.or.kr).

## Abbreviations

AML: Acute myeloid leukemia
BMI: Body mass index
COPD: Chronic obstructive pulmonary disease
CI: Confidence intervals
HR: Hazard ratio
ICS: Inhaled corticosteroid
NHIS-NSC: National Health Insurance Service–National Sample Cohort
LABA: Long-acting beta-2 agonist
LAMA: Long-acting muscarinic antagonist
PDE-4 inhibitor: Phosphodiesterase-4 inhibitor
SABA: Short-acting beta-2 agonist
SAMA: Short-acting muscarinic antagonist
