# Supplementary material for: Cancer development in patients with COPD: a retrospective analysis of the National Health Insurance Service-National Sample Cohort in Korea
Source: BMC Pulm Med. 2020 Jun 15;20:170. doi: 10.1186/s12890-020-01194-8 (PMC7296952; doi:10.1186/s12890-020-01194-8)
Supplement: Supplementary file 1 — Additional file 1. Table 1. Risk factors for the development of stomach cancer. Table 2. Risk factors for the development of colorectal cancer. Table 3. Risk factors for the development of liver cancer. Table 4. Risk factors for the development of esophageal cancer. Table 5. Risk factors for the development of bladder cancer. [file 12890_2020_1194_MOESM1_ESM.docx]

**Table 1.** Risk factors for the development of stomach cancer.

|  | Cox regression analysis | | | |
| --- | --- | --- | --- | --- |
|  | Univariate  Hazard ratio ( 95% CI) | p-value | Multivariate  Hazard ratio ( 95% CI) | p-value |
| Age (years) | 1.052 (1.051-1.054) | <0.001 | 1.057 (1.055-1.059) | <0.001 |
| Male (vs female) | 1.929 (1.861-1.999) | <0.001 | 2.009 (1.927-2.095) | <0.001 |
| BMI (kg/m^2^) |  | <0.001 |  | <0.001 |
| <20 | 1.307 (1.233-1.386) |  | 1.149 (1.085-1.218) |  |
| 20 ≤ <25 | Reference |  | Reference |  |
| 25 ≤ <30 | 1.005 (0.893-1.132) |  | 1.010 (0.895-1.140) |  |
| 30 ≤ | 0.910 (0.764-1.084) |  | 1.015 (0.852-1.210) |  |
| History of hypertension | 1.340 (1.270-1.414) | <0.001 | 0.989 (0.935-1.045) | 0.689 |
| History of diabetes mellitus | 1.466 (1.366-1.574) | <0.001 | 1.137 (1.058-1.222) | <0.001 |
| COPD diagnosis | 1.692 (1.549-1.849) | <0.001 | 1.056 (0.966-1.155) | 0.231 |
| Exercise level |  |  |  | <0.001 |
| Never Exercise | 1.031 (0.986-1.078) |  | 1.074 (1.028-1.123) |  |
| 1-2 times a week | 0.894 (0.820-0.974) |  | 0.981 (0.899-1.070) |  |
| ≥ 3 times a week | Reference |  | Reference |  |
| Smoking status |  | <0.001 |  | <0.001 |
| Never smoker | Reference |  | Reference |  |
| Former smoker | 1.413 (1.337-1.493) |  | 1.111 (1.048-1.178) |  |
| Current smoker | 1.501 (1.446-1.557) |  | 1.235 (1.184-1.289) |  |

Definition of abbreviations : BMI = body mass index; COPD = chronic obstructive pulmonary disease, * = statistically significant hazard ratio (p-value <0.01).

**Table 2.** Risk factors for the development of colorectal cancer.

|  | Cox regression analysis | | | |
| --- | --- | --- | --- | --- |
|  | Univariate  Hazard ratio ( 95% CI) | p-value | Multivariate  Hazard ratio ( 95% CI) | p-value |
| Age (years) | 1.047 (1.045 - 1.048) | <0.001 | 1.047 (1.046-1.049) | <0.001 |
| Male (vs female) | 1.329 (1.291 - 1.368) | <0.001 | 1.43 (1.382-1.48) | <0.001 |
| BMI (kg/m^2^) |  | <0.001 |  | 0.003 |
| <20 | 1.135 (1.076-1.197) |  | 1.045 (0.991-1.102) |  |
| 20 ≤ <25 | Reference |  | Reference |  |
| 25 ≤ <30 | 1.042 (0.935-1.161) |  | 1.025 (0.920-1.142) |  |
| 30 ≤ | 1.134 (0.980-1.312) |  | 1.153 (0.996-1.334) |  |
| History of hypertension | 1.500 (1.435 - 1.567) | <0.001 | 1.096 (1.047-1.147) | <0.001 |
| History of diabetes mellitus | 1.580 (1.490 - 1.676) | <0.001 | 1.233 (1.162-1.309) | <0.001 |
| COPD diagnosis | 1.882 (1.750 - 2.023) | <0.001 | 1.273 (1.183-1.37) | <0.001 |
| Exercise level |  | <0.001 |  | 0.191 |
| Never Exercise | 1.001 (0.964-1.039) |  | 1.014 (0.978-1.052) |  |
| 1-2 times a week | 0.886 (0.824-0.953) |  | 0.981 (0.911-1.055) |  |
| ≥ 3 times a week | Reference |  | Reference |  |
| Smoking status |  | <0.001 |  | <0.001 |
| Never smoker | Reference |  | Reference |  |
| Former smoker | 1.151 (1.096 - 1.209) |  | 1.046 (0.992-1.102) |  |
| Current smoker | 1.140 (1.103 - 1.178) |  | 1.088 (1.047-1.13) |  |

Definition of abbreviations : BMI = body mass index; COPD = chronic obstructive pulmonary disease, * = statistically significant hazard ratio (p-value <0.01).

**Table 3.** Risk factors for the development of liver cancer.

|  | Cox regression analysis | | | |
| --- | --- | --- | --- | --- |
|  | Univariate  Hazard ratio ( 95% CI) | p-value | Multivariate  Hazard ratio ( 95% CI) | p-value |
| Age (years) | 1.025 (1.024 - 1.027) | <0.001 | 1.027 (1.025 - 1.028) | <0.001 |
| Male (vs female) | 1.603 (1.559 - 1.649) | <0.001 | 1.621 (1.568 - 1.677) | <0.001 |
| BMI (kg/m^2^) |  | <0.001 |  | <0.001 |
| <20 | 1.105 (1.050-1.162) |  | 1.048 (0.997-1.102) |  |
| 20 ≤ <25 | Reference |  | Reference |  |
| 25 ≤ <30 | 1.027 (0.926-1.138) |  | 1.015 (0.916-1.124) |  |
| 30 ≤ | 1.170 (1.019-1.344) |  | 1.232 (1.073-1.414) |  |
| History of hypertension | 1.210 (1.157 - 1.266) | <0.001 | 1.019 (0.972 - 1.068) | 0.437 |
| History of diabetes mellitus | 1.592 (1.506 - 1.683) | <0.001 | 1.389 (1.313 - 1.470) | <0.001 |
| COPD diagnosis | 1.539 (1.428 - 1.659) | <0.001 | 1.217 (1.128 - 1.313) | <0.001 |
| Exercise level |  | <0.001 |  | <0.001 |
| Never Exercise | 1.052 (1.014-1.090) |  | 1.106 (1.067-1.147) |  |
| 1-2 times a week | 0.976 (0.911-1.046) |  | 1.006 (0.938-1.078) |  |
| ≥ 3 times a week | Reference |  | Reference |  |
| Smoking status |  | <0.001 |  | <0.001 |
| Never smoker | 1 |  | 1 |  |
| Former smoker | 1.267 (1.21 - 1.327) |  | 1.040 (0.990 - 1.093) |  |
| Current smoker | 1.392 (1.351 - 1.435) |  | 1.162 (1.122 - 1.204) |  |

Definition of abbreviations : BMI = body mass index; COPD = chronic obstructive pulmonary disease, * = statistically significant hazard ratio (p-value <0.01).

**Table 4.** Risk factors for the development of esophageal cancer.

|  | Cox regression analysis | | | |
| --- | --- | --- | --- | --- |
|  | Univariate  Hazard ratio ( 95% CI) | p-value | Multivariate  Hazard ratio ( 95% CI) | p-value |
| Age (years) | 1.076 (1.070 - 1.082) | <0.001 | 1.084 (1.077 - 1.091) | <0.001 |
| Male (vs female) | 4.123 (3.527- 4.819) | <0.001 | 3.599 (3.020 - 4.288) | <0.001 |
| BMI (kg/m^2^) |  | <0.001 |  | <0.001 |
| <20 | 2.062 (1.745 - 2.436) |  | 1.605 (1.353 - 1.904) |  |
| 20 ≤ <25 | Reference |  | Reference |  |
| 25 ≤ <30 | 0.732 (0.513 - 1.045) |  | 0.785 (0.544 - 1.133) |  |
| 30 ≤ | 0.336 (0.155-0.731) |  | 0.446 (0.204 - 0.977) |  |
| History of hypertension | 1.400 (1.160 - 1.688) | <0.001 | 1.058 (0.871 - 1.285) | 0.570 |
| History of diabetes mellitus | 1.542 (1.204 - 1.974) | <0.001 | 1.136 (0.884 - 1.459) | 0.319 |
| COPD diagnosis | 1.941 (1.445 - 2.607) | <0.001 | 0.944 (0.700 - 1.274) | 0.708 |
| Exercise level |  | 0.031 |  | 0.470 |
| Never Exercise | 0.988 (0.847 - 1.152) |  | 1.010(0.865 - 1.179) |  |
| 1-2 times a week | 0.814 (0.600 - 1.105) |  | 0.917(0.672 - 1.251) |  |
| ≥ 3 times a week | Reference |  | Reference |  |
| Smoking status |  | <0.001 |  | <0.001 |
| Never smoker | Reference |  | Reference |  |
| Former smoker | 1.998 (1.643 - 2.431) |  | 1.304 (1.062 - 1.601) |  |
| Current smoker | 2.852 (2.515 - 3.235) |  | 1.968 (1.709 - 2.267) |  |

Definition of abbreviations : BMI = body mass index; COPD = chronic obstructive pulmonary disease, * = statistically significant hazard ratio (p-value <0.01).

**Table 5.** Risk factors for the development of bladder cancer.

|  | Cox regression analysis | | | |
| --- | --- | --- | --- | --- |
|  | Univariate  Hazard ratio ( 95% CI) | p-value | Multivariate  Hazard ratio ( 95% CI) | p-value |
| Age (years) | 1.057 (1.054 - 1.060) | <.0001 | 1.06 (1.077 - 1.091) | <0.001 |
| Male (vs female) | 1.785 (1.671 - 1.907) | <.0001 | 1.929 (1.785 - 2.083) | <0.001 |
| BMI (kg/m^2^) |  | 0.1025 |  | 0.800 |
| <20 | 1.144 (1.019 - 1.284) |  | 1.029 (0.916 - 1.156) |  |
| 20 ≤ <25 | Reference |  | Reference |  |
| 25 ≤ <30 | 1.043 (0.825 - 1.320) |  | 1.033 (0.813 - 1.312) |  |
| 30 ≤ | 0.962 (0.691 - 1.341) |  | 1.034 (0.739 - 1.446) |  |
| History of hypertension | 1.640 (1.495 - 1.800) | <0.001 | 1.167 (1.060 - 1.286) | 0.002 |
| History of diabetes mellitus | 1.460 (1.277-1.669) | <0.001 | 1.068 (0.932 - 1.223) | 0.345 |
| COPD diagnosis | 1.842 (1.569 - 2.163) | <0.001 | 1.146 (0.9074 - 1.348) | 0.101 |
| Exercise level |  | <0.001 |  | 0.154 |
| Never Exercise | 0.906 (0.837 - 0.980) |  | 0.947 (0.873 - 1.027) |  |
| 1-2 times a week | 0.810 (0.692 - 0.948) |  | 0.911 (0.776 - 1.070) |  |
| ≥ 3 times a week | Reference |  | Reference |  |
| Smoking status |  | <0.001 |  | 0.005 |
| Never smoker | Reference |  | Reference |  |
| Former smoker | 1.364 (1.231 - 1.512) |  | 1.089 (0.976 - 1.215) |  |
| Current smoker | 1.323 (1.233 - 1.421) |  | 1.141 (1.053 - 1.237) |  |

Definition of abbreviations : BMI = body mass index; COPD = chronic obstructive pulmonary disease, * = statistically significant hazard ratio (p-value <0.01).

.
